# Sporotrichosis Caused by Non-Wild Type *Sporothrix brasiliensis* Strains

**DOI:** 10.3389/fcimb.2022.893501

**Published:** 2022-05-27

**Authors:** Andréa Reis Bernardes-Engemann, Gabriela Ferreira Tomki, Vanessa Brito de Souza Rabello, Fernando Almeida-Silva, Dayvison Francis Saraiva Freitas, Maria Clara Gutierrez-Galhardo, Rodrigo Almeida-Paes, Rosely Maria Zancopé-Oliveira

**Affiliations:** ^1^Laboratório de Micologia, Instituto Nacional de Infectologia Evandro Chagas, Fundação Oswaldo Cruz, Rio de Janeiro, Brazil; ^2^Laboratório de Pesquisa Clínica em Dermatologia Infecciosa - Instituto Nacional de Infectologia Evandro Chagas – Fundação Oswaldo Cruz, Rio de Janeiro, Brazil

**Keywords:** sporotrichosis, *Sporothrix brasiliensis*, antifungal drugs, antifungal susceptibility, non-wild type strain, treatment

## Abstract

The zoonotic transmission of sporotrichosis due to *Sporothrix brasiliensis* occurs largely in Rio de Janeiro state, Brazil since the 1990´s. Most patients infected with *S. brasiliensis* respond well to itraconazole or terbinafine. However, a few patients have a slow response or do not respond to the treatment and develop a chronic infection. The aim of this study was to analyze strains of *S. brasiliensis* against five different drugs to determine minimal inhibitory concentration distributions, to identify non-wild type strains to any drug evaluated and the clinical aspects of infections caused by them. This study evaluated 100 *Sporothrix* spp. strains obtained from 1999 to 2018 from the Evandro Chagas National Institute of Infectious Diseases, Fiocruz, which were identified through a polymerase chain reaction using specific primers for species identification. Two-fold serial dilutions of stock solutions of amphotericin B, itraconazole, posaconazole, ketoconazole and terbinafine prepared in dimethyl sulfoxide were performed to obtain working concentrations of antifungal drugs ranging from 0.015 to 8.0 mg/L. The broth microdilution reference method was performed according the M38-A2 CLSI guideline. All strains were identified as *S. brasiliensis* and thirteen were classified as non-wild type, two of them against different drugs. Non-wild type strains were identified throughout the entire study period. Patients infected by non-wild type strains presented prolonged treatment times, needed increased antifungal doses than those described in the literature and one of them presented a permanent sequel. In addition, three of them, with immunosuppression, died from sporotrichosis. Despite the broad use of antifungal drugs in hyperendemic areas of sporotrichosis, an emergence of non-wild type strains did not occur. The results of *in vitro* antifungal susceptibility tests should guide sporotrichosis therapy, especially in immunosuppressed patients.

## 1 Introduction

Sporotrichosis is the most reported and globally distributed subcutaneous mycosis (Chakrabarti et al., 2015). Its etiologic agent, for more than a century, was reported to be the single species *Sporothrix schenckii* (Lopes-Bezerra et al., 2006), but after phenotypic and molecular studies (Marimon et al., 2007), it was discovered that human sporotrichosis has three major agents: *S. schenckii*, *Sporothrix brasiliensis* and *Sporothrix globosa* (Chakrabarti et al., 2015). All these species are thermodimorphic fungi that present in a filamentous form in nature or when cultured at 25 – 30°C, and in a yeast-like form in parasitism or when cultured at 35 – 37°C in enriched culture media (de Lima Barros et al., 2011a).

Sporotrichosis has two major transmission forms. The classic sapronotic transmission occurs when the patient has a skin trauma with an environmental material harboring the fungus, such as wood (Quintal, 2000), rose thorns (Kieselova et al., 2017), hay (Dhingra et al., 2015), corn stalks (Mehta et al., 2007) or *Sphagnum* moss (Hajjeh et al., 1997). This transmission form was recognized since the description of the disease and, for long time, sporotrichosis was described as an occupational infection (Lopes-Bezerra et al., 2006). The zoonotic transmission occurs when the patient is injured by an animal, especially naturally infected domestic cats. The zoonotic transmission occurs largely in Rio de Janeiro state, Brazil, since late 1990´s (Barros et al., 2001; Orofino-Costa et al., 2017), but, in last years, this transmission form is spreading to other Brazilian states (de Oliveira Bento et al., 2021; Rabello et al., 2022) and South American countries (Etchecopaz et al., 2020; Rossow et al., 2020). The major agent in Brazilian zoonotic sporotrichosis is *S. brasiliensis* (Rabello et al., 2022), while the classic transmission form is usually related to *S. schenckii* and *S. globosa* (Orofino-Costa et al., 2017). An additional rare transmission form of this mycosis is through the inhalation of fungal cells, leading to primary pulmonary sporotrichosis (Rohatgi, 1980).

The first drug choice for sporotrichosis treatment is itraconazole, with terbinafine and potassium iodide as alternatives (Orofino-Costa et al., 2017). Amphotericin B is indicated in severe disease cases or when unresponsive to the other drugs (Kauffman et al., 2007). Most patients infected with *S. brasiliensis* respond well to these drugs (de Lima Barros et al., 2011b), although therapeutic failures have been reported (Almeida-Paes et al., 2017b; Belda et al., 2021). In the last decade, epidemiologic cutoff values (ECV) were established for *S. brasiliensis* and *S. schenckii* to detect non-wild type strains against some antifungal drugs (Espinel-Ingroff et al., 2017). Non-wild type strains have high chances to present mechanisms of acquired or mutational resistance to the tested antifungal drug (Espinel-Ingroff and Turnidge, 2016). The aim of the present study was to analyze *S. brasiliensis* strains isolated throughout 20 years of hyperendemic zoonotic sporotrichosis in Rio de Janeiro, Brazil, against five different drugs to determine their minimal inhibitory concentration distributions, to identify non-wild type strains to any evaluated drug and to describe the clinical aspects of infections caused by them.

## 2 Materials and methods

### 2.1 Fungal Strains

#### 2.1.1* Sporothrix* spp. Strains

A total of 100 *Sporothrix* spp. strains, obtained from 1999 to 2018 and stored at -80°C in the Mycology Laboratory from the Evandro Chagas National Institute of Infectious Diseases, Fiocruz, was evaluated in this study. All strains were isolated from human patients with sporotrichosis. These strains were randomly chosen from a collection of 1,226 *Sporothrix* spp. isolated at our institution since 1998.

#### 2.1.2 Control Strains

The strains *Aspergillus fumigatus* (ATCC 204305) and *Aspergillus flavus* (ATCC 204304) were used as quality controls of the antifungal susceptibility assays. The strains *S. brasiliensis* (CFP 00722), *S. schenckii* (CFP 00448) and *S. globosa* (CFP 01021) were used as controls of the molecular test of identification. These three *Sporothrix* strains were previously identified by the gold standard method for *Sporothrix* identification, that is the partial sequencing of the calmodulin gene (Marimon et al., 2007).

### 2.2 Molecular Identification

The strains were identified through a polymerase chain reaction (PCR) for specific *Sporothrix* spp. identification (Rodrigues et al., 2015). The DNA was extracted directly from the mycelial *Sporothrix* colonies after seven days of growth on Potato Dextrose Agar (PDA) medium at 25°C. Cell lysis was performed as previously described (Muniz et al., 2010) with minor adaptations, such as the use of TBE buffer (1M Tris pH 8, 50 mM EDTA, 20% sucrose) and DNA precipitation with isopropanol. The isolates were identified by a species-specific PCR. In summary, the following primers for the three major pathogenic *Sporothrix* species were used: Sbra-F (5’ – CCC CCG TTT GAC GCT TGG – 3’) and Sbra-R (5’ – CCC GGA TAA CCG TGT GTC ATA AT – 3’) for *S. brasiliensis*; Ssch-F (5 – TTT CGA ATG CGT TCG GCT GG – 3’) and Ssch-R (5’ – CTC CAG ATC ACC GTG TCA – 3’) for *S. schenckii;* and Sglo-F (5’ – CGC CTA GGC CAG ATC ACC ACT AAG – 3’) and Sglo-R (5’ – CCA ATG TCT ACC CGT GCT – 3’) for *S. globosa*. PCR conditions were previously described (Rodrigues et al., 2015) and amplicon sizes were estimated after electrophoresis on 1.0% agarose gels using a 1 kb molecular weight marker.

### 2.3 Antifungal Susceptibility Assay

The broth microdilution reference method was performed according the M38-A2 CLSI guideline (CLSI, 2008). Stock solutions of itraconazole (ITR), posaconazole (POS), ketoconazole (KET), terbinafine (TRB) and amphotericin B (AMB), all from Sigma-Aldrich Corporation (San Luis, MO, USA) were prepared in dimethyl sulfoxide at 16 µg/mL. From the stock solutions, two-fold dilutions were performed to obtain the antifungal final concentrations, which ranged from 8 to 0.015 µg/mL in RPMI-1640 with phenol red, without sodium bicarbonate buffered with 3-(N-Morpholino)propanesulfonic acid (MOPS) at pH 7.0. In 96-well polystyrene round bottom microplates, 100 µL of the medium with the different antifungal concentrations were added to the wells. Inocula of 1 to 5×10^4^ conidia/mL were prepared after *Sporothrix* inoculation on PDA and subsequent incubation for seven days at 35°C. Fungal conidia suspension (100 µL) in RPMI-1640 buffered with MOPS were then added to each well-containing the drug dilutions. Positive controls consisted of 100 µL of conidia suspension with 100 µL of RPMI-1640 buffered with MOPS and without any antifungal drug, to assess regular fungal growth. Negative controls consisted of 200 µL of RPMI-1640 buffered with MOPS and without any antifungal drug, to confirm the sterility of the culture medium. Plates were incubated at 35°C for 48-72 hours and the minimal inhibitory concentration (MIC) was determined visually by comparison with growth and growth-free wells (positive and negative controls, respectively). For AMB, ITR and POS, the MIC endpoints were the lowest concentrations that completely inhibited fungal growth. For KET, the MIC was the lowest concentration that resulted in a 50% reduction in growth relative to that of the growth control, and, for TRB, it was the lowest concentration that resulted in at least an 80% reduction in growth relative to the control without the antifungal drug. Antifungal susceptibility tests were performed at least twice and were validated by the determination of the same MICs in different experiments (CLSI, 2008; Espinel-Ingroff et al., 2017).

### 2.4 Classification of Non-Wild Type Strains

The ECV proposed in an international study for *S. brasiliensis* and AMB, ITR, POS, KET and TRB were used (Espinel-Ingroff et al., 2017). Strains with MIC values for AMB above 4.0 µg/mL, ITR, POS and KET above 2.0 µg/mL, and TRB above 0.12 µg/mL were classified as non-wild type strains.

### 2.5 Analyses of MIC Results

GraphPad Prism 7 was used for the analyses. Descriptive statistics were made to obtain the MIC range, MIC50 and MIC90 values, and geometric means of each antifungal drug. The MIC50 and MIC90 values are the concentration of the antifungal drug capable to inhibit the growth of 50% and 90% of all studied *Sporothrix* strains, respectively. The Kruskal–Wallis one-way analysis of variance with the Dunn’s multiple comparison test was used to compare MIC values during four divisions of the study period: 1999 to 2003, 2004 to 2008, 2009 to 2013 and 2014 to 2018. The Mann-Whitney test was used to compare MIC values of wild type and non-wild type strains. A value of P < 0.05 was considered significant.

### 2.6 Patient Information

The use of patient data was approved by the Research Ethics Committee of the Evandro Chagas National Institute of Infectious Diseases (CAAE 16160619.5.0000.5262). All patient data were assessed anonymously. The medical records of the patients infected with a strain classified as non-wild type for any of the studied antifungal drug, regardless of its use during patient treatment were reviewed. The following variables were studied: sex, age, year of fungal isolation, transmission form, clinical form, presence of comorbidities, treatment, and outcome. Alcoholism was defined using the CAGE questionnaire (Ewing, 1984). The last follow-up of patients occurred in December 2021.

## 3 Results

### 3.1 MIC Values of *S. brasiliensis* During the Hyperendemic Sporotrichosis in Rio de Janeiro, Brazil

All strains were identified as *S. brasiliensis* by the species-specific PCR, that is, only the primer pair SbraF and SbraR yielded amplicons of 469 bp. **Table 1** summarizes the *in vitro* antifungal susceptibility of these 100 *S. brasiliensis* strains to five antifungal drugs. As expected, terbinafine was the antifungal drug with the lowest MIC 50 and geometric mean values, while amphotericin B presented the highest MIC parameters among the five studied antifungal drugs. The number of strains presenting MIC values higher than the MIC90 were 9 for KET, 6 for ITR, AMB and TRB, and 1 to POS. According to the Kruskal–Wallis one-way analysis of variance with the Dunn’s multiple comparison test, there was no statistically supported difference in the *in vitro* antifungal susceptibility of these drugs in the four quinquennials studied.

**Table 1:** Descriptive summary of minimal inhibitory concentrations (µg/mL) of five antifungal drugs against 100 *S. brasiliensis* strains from Rio de Janeiro, Brazil.

Antifungal drug	Range		MIC 50	MIC 90	Geometric mean
	Minimum	Maximum			
Itraconazole	0.12	8.0	0.5	1.0	0.4186
Terbinafine	<0.015	0.5	0.015	0.03	0.0249
Amphotericin B	0.015	8.0	0.5	1.0	0.5352
Posaconazole	0.015	4.0	0.25	0.5	0.1969
Ketoconazole	0.015	8.0	0.12	0.5	0.1295

### 3.2 Sporothrix brasiliensis Non-Wild Type Strains

**Figure 1:**
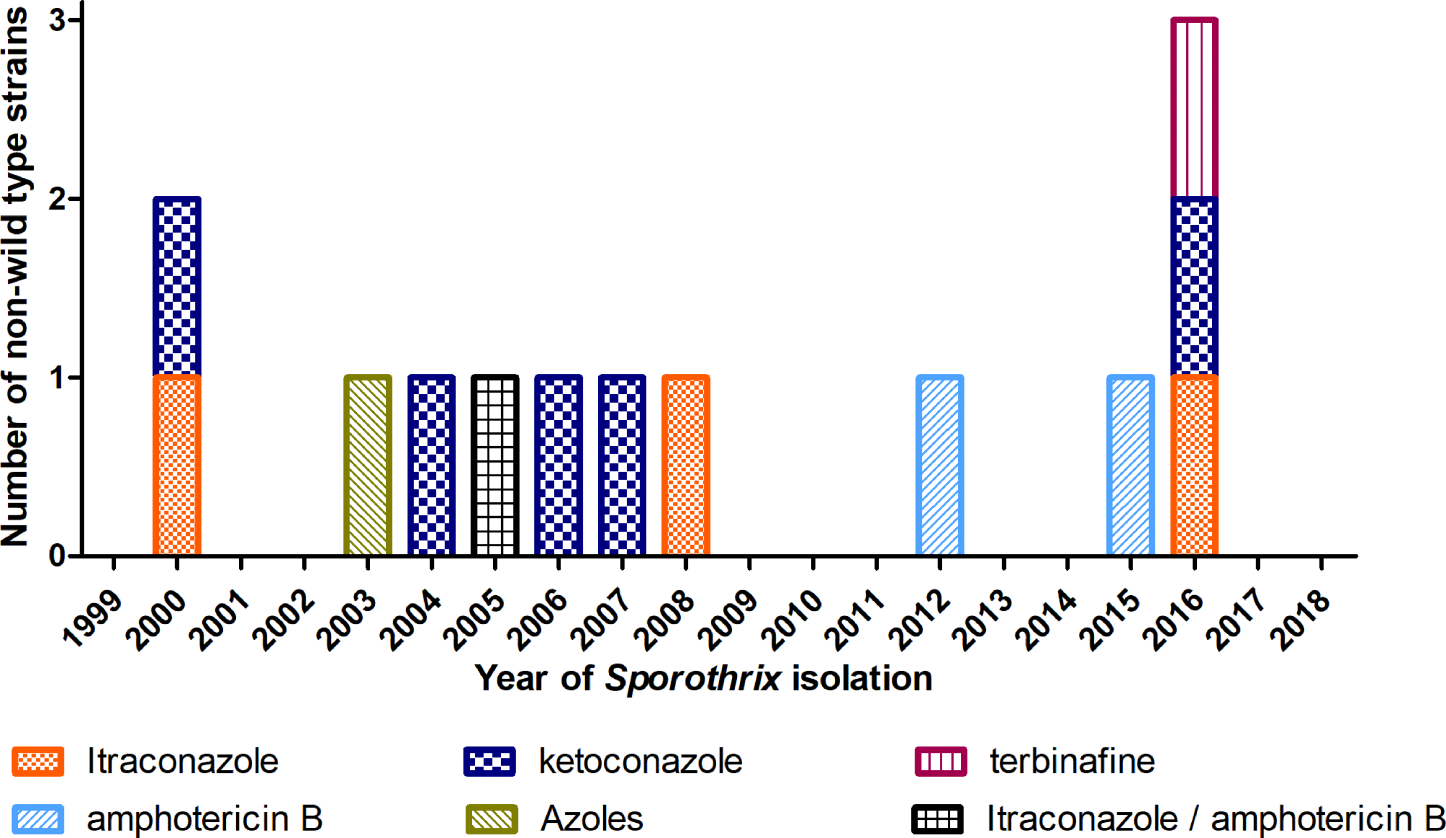
*Sporothrix brasiliensis* non-wild type strains during 20 years of hyperendemic zoonotic sporotrichosis in Rio de Janeiro, Brazil.

According to the established ECV, 13 strains were classified as non-wild type against at least one antifungal drug included in this study. We identified non-wild type strains to KET (n = 6), ITR (n = 5), AMB (n = 3), POS (n = 1) and TRB (n = 1). Two strains were non-wild type to more than one antifungal drug: one to KET, POS and ITR and another to ITR and AMB. Non-wild type strains were identified through 2000 to 2016, as depicted in **Figure 1**. **Table 2** presents the *in vitro* antifungal susceptibility of these 13 *S. brasiliensis* strains to the five antifungal drugs. It is possible to see that the geometric mean of MIC of most antifungal drugs are slightly higher than those observed in **Table 1**: 0.9451 for ITR, 0.0141 for TRB, 0.7937 for AMB, 0.3326 for POS and 0.5776 for KET. The comparison of MIC values from wild type (n = 87) and non-wild type strains (n = 13) revealed a significant difference for ITR (P = 0.027) and KET (P = 0.009).

**Table 2:** Minimal inhibitory concentrations (µg/mL) of five antifungal drugs against *S. brasiliensis* non-wild type strains from Rio de Janeiro, Brazil.

Case	Itraconazole	Terbinafine	Amphotericin B	Posaconazole	Ketoconazole
1	4.0	0.015	0.5	0.5	0.25
2	0.5	0.03	0.5	0.5	8.0
3	8.0	0.015	0.5	4.0	4.0
4	1.0	<0.015	0.5	0.25	4.0
5	4.0	<0.015	8.0	0.25	0.25
6	0.25	<0.015	0.5	0.25	4.0
7	0.5	<0.015	0.5	0.25	4.0
8	4.0	<0.015	0.5	0.25	0.12
9	0.5	<0.015	8.0	0.25	0.12
10	0.25	<0.015	8.0	0.25	0.03
11	0.12	0.5	0.5	0.25	0.06
12	0.25	<0.015	0.5	0.12	4.0
13	4.0	0.015	0.5	0.25	0.06

### 3.3 Sporotrichosis Caused by Non-Wild Type Strains

**Table 3** summarizes the demographic, epidemiological, clinical, and therapeutic aspects, as well as the outcome of patients infected with the 13 non-wild type *S. brasiliensis* strains identified in the present study. Ten patients needed more than six months of treatment: one patient (case 4) was cured after six months with ITR; another patient (case 8) presented clinical cure after four months of treatment with TRB but had a relapse of infection three months later; and another patient (case 12) was treated with ITR and AMB for four months but died from HIV complications.

**Table 3:** Demographic, epidemiological, clinical, and therapeutic aspects, and outcome of patients with non-wild type *Sporothrix brasiliensis* strains.

Case	Sex/age (years)	Year of *Sporothrix* isolation	Transmission	Clinical Form	Comorbidity/Immunosuppression	nWT	Treatment		Outcome
							Antifungal	Months	
**1**	F/54	2000	Cat scratch	LC	NA	ITR	ITR up to 400 mg/day	9	Loss of follow-up
**2**	F/63	2000	Cat bite	DC	None	KET	ITR up to 400 mg/day, FLU 200 mg/day, curettage	9	Cure
**3**	M/18	2003	Contact with diseased cat and soil/plants	Disseminated	Alcoholism	ITR POS KET	ITR up to 400 mg/day, AMB 8,885 mg	37	Cure
**4**	F/34	2004	Cat scratch	LC	HBP and obesity	KET	ITR 100 mg/day	6	Cure
**5**	F/66	2005	Contact with cat	LC	None	ITR AMB	ITR 100 mg/day, cryosurgery, curettage	14	Cure
**6**	M/60	2006	Cat scratch	DC	Stroke sequelae	KET	ITR 100 mg/day, cryosurgery, curettage	15	Cure
**7**	M/68	2007	Cat bite and scratch	LC	HBP	KET	TRB up to 500 mg/day, ITR up to 200 mg/day, cryosurgery, curettage	38	Cure
8^*^	F/34	2008	Cat bite and scratch	DC	None	ITR	TRB 250 mg/day	4	Cure
**9**	M/45	2012	No history	Disseminated	HIV, alcoholism	AMB	ITR up to 200 mg/day, AMB 11,420 mg	16	Death
**10**	M/43	2015	Contact with cat	Disseminated	Renal transplant	AMB	ITR 200 mg/day, AMB	8	Death
**11**	F/73	2016	Cat scratch	LC and EC unifocal (bone and tendon)	DM, HBP	TRB	ITR up to 400 mg/day, TRB 250 mg/day	34	Cure (Amputation of 4^th^ left finger)
**12**	M/29	2016	Cat scratch	Disseminated	HIV	KET	ITR 200 mg/day, AMB 2,000 mg	4	Death
13^**^	F/73	2016	Cat scratch	DC (leg and hand)	HBP	ITR	ITR 100 mg/day	84	Treating

nWT, non-wild type drug; M, male; F, female; DC, disseminated cutaneous form; LC, lymphocutaneous form; EC, extracutaneous form; DM, diabetes mellitus; HBP, high blood pressure; HIV, human immunodeficiency virus; ITR, itraconazole; TRB, terbinafine; AMB, amphotericin B; FLC, fluconazole; *Relapse 3 months later and treated with TRB 250 mg for 1 month. **Two episodes of previous cutaneous sporotrichosis 10 years before. NA, not available.

## 4 Discussion

Sporotrichosis has emerged as an important mycosis in several countries, such as China, India, Mexico and Brazil (Chakrabarti et al., 2015). Although most patients have a good response to the first-choice drug for treatment (Freitas et al., 2010) and to other therapeutic alternatives (Francesconi et al., 2009; Macedo et al., 2015), it is still a challenge to treat sporotrichosis in some patients, especially those with comorbidities or those with immunossuppresion (Freitas et al., 2014; Fichman et al., 2021). In last years, an international, multicenter study defined ECV values for *S. brasiliensis* and *S. schenckii* to several antifungal drugs that may be used in the sporotrichosis treatment (Espinel-Ingroff et al., 2017). According to this study, these ECV should help clinicians to identify cases caused by strains unlikely to present a good response to therapy. To the best of our knowledge, this is the first study describing clinical aspects of a series of human patients infected with non-wild type *S. brasiliensis* strains.

The MIC values obtained in this study is in accordance with other studies that report the high *in vitro* activity of TRB and a lower anti-*Sporothrix* activity of AMB (Marimon et al., 2008; Borba-Santos et al., 2015; Almeida-Paes et al., 2017a). We were not able to establish any temporal relationship of the MIC values for the drugs herein studied. A previous study that compared susceptibilities of *S. brasiliensis* isolated before 2004 with isolates from 2011 – 2012 showed that, when the CLSI M38-A2 protocol was used, POS susceptibility of newer strains were higher than that observed for older strains, while AMB, ITR and TRB susceptibilities were similar, regardless the year of isolation of the strains (Borba-Santos et al., 2015), which support most of the results of this work. The temporal differences observed in POS susceptibility in the two studies may be related to the number or origin of studied strains.

The non-wild type strains occurred throughout the study period. These strains are supposed to have mechanisms of antifungal resistance. However, it is not possible to know, with the results of the present study, if the strains truly have acquired resistance and the mechanism of such resistance. In previous studies, we have shown that prolonged sporotrichosis treatment, with high doses of antifungal drugs do not lead to an emergence of antifungal resistance (Almeida-Paes et al., 2017b; Cruz et al., 2021). Until 2004, 759 human cases were diagnosed and treated in Rio de Janeiro, Brazil (Schubach et al., 2008). Despite this low number, four non-wild type strains were identified in the first five years of the hyperendemic zoonotic sporotrichosis in Rio de Janeiro, which reinforces the theory that high antifungal usage in several human patients does not play a role in the emergence of *Sporothrix* resistance. Another aspect that supports this theory is that, for most patients in this study, the *S. brasiliensis* strain was isolated at diagnosis, before the beginning of antifungal treatment, indicating that the patients were infected by a non-wild type strain. It is well known that the use of agricultural fungicides may lead to the emergence of resistant *Aspergillus fumigatus* strains (Doughty et al., 2021). Sporotrichosis caused by *S. brasiliensis* is an urban public health problem, usually related to low per capita incomes and insufficient supply of potable water (Alzuguir et al., 2020). Whether the water quality may be associated with the appearance of resistant strains remains to be elucidated.

The last guideline for diagnosis and management of endemic mycoses recommends that patients with cutaneous forms of sporotrichosis should be treated with ITR 200 mg/day or TRB 500 mg/day for three to six months, while those with disseminated forms should be treated with AMB (Thompson et al., 2021). This is also recommended by the specific sporotrichosis guideline proposed by the Infectious Diseases Society of America (Kauffman et al., 2007). Some studies from our group in the hyperendemic area of zoonotic sporotrichosis caused by *S. brasiliensis*, which differs from most other areas where sporotrichosis is endemic, show that the mean time of sporotrichosis treatment with ITR (100 mg/day) and TRB (250 mg/day) is around 12 weeks (de Lima Barros et al., 2011b; Francesconi et al., 2011), with clinical cure rates ranging from 89 to 94.6% (Freitas et al., 2010; de Lima Barros et al., 2011b). It is important to note that the majority of patients infected with non-wild type strains (n = 10; 76.9%) needed more than the recommended three to six months of treatment. The remaining three patients needed more than 12 weeks of therapy, which is the mean treatment time at our institution. The refractoriness of four patients (cases 3, 9, 10 and 12) could be explained by the disseminated clinical form of the disease associated with HIV, transplant and alcoholism, which usually require prolonged treatment (Freitas et al., 2014; Fichman et al., 2021), three of them (9,10 and 12) progressed to death. On the other hand, four patients (cases 4, 5, 6, and 8) remained with the lowest dose of the antifungal. Patient case 13 is a chronic case, of apparent cure and relapses for years, with a low compliance to treatment, continuous exposition to sick cats, and does not tolerate augmentation of doses or the adjuvant cryosurgery sessions. These various regimens reflect the change in clinical protocols over the 20 years of this series. Moreover, we know that the ITR pharmacokinetics is erratic. Unfortunately, dosage of serum ITR concentrations, which would help about therapeutic doses, is not available at our institution, and in most health centers where sporotrichosis is endemic. In the group without immunosuppression, all were cured, except case 11, whose cure was associated with a serious sequela, amputation; and in case 13, attributed to a low compliance. For both cases, immunosenescence may play a role. For at least three patients, with cutaneous forms and without comorbidities or immunosuppression, the only reasonable explanation for the prolonged treatment was the infection by a non-wild type strain.

Interestingly, some of the patients herein studied were infected by a non-wild type strain for one antifungal drug, but they were treated with a different drug. The use of another antifungal drug to treat the infection caused by a non-wild type strain did not reduce in the duration of therapy, indicating that cross-resistance mechanisms are likely to occur in *S. brasiliensis*. For example, five patients were infected by non-wild type strains only to KET, but were treated with other drugs, including azoles. Although oral KET is not recommended to treat sporotrichosis due to its adverse effects, *in vitro* testing of KET susceptibility may predict treatment failure with other azoles.

In conclusion, sporotrichosis caused by *S. brasiliensis* non-wild type strains may be of difficult management, needing increased antifungal dosages and prolonged treatment than that described in the literature. However, it does not seem to be a frequent event over time. *In vitro* antifungal testing of *Sporothrix* strains may predict treatment failures in human sporotrichosis, which also occurs in cat sporotrichosis (Nakasu et al., 2021). This approach is extremely important in immunosuppressed patients, in whom sporotrichosis is a cause of hospitalization and death (Falcão et al., 2020). Further studies on resistance mechanisms of *Sporothrix* strains and on the discovery of new antifungal drugs, natural products with anti-*Sporothrix* activity or drug repurposing may help in the management of refractory cases of sporotrichosis.

## Data Availability Statement

The original contributions presented in the study are included in the article/**Supplementary Material**. Further inquiries can be directed to the corresponding author.

## Ethics Statement

The studies involving human participants were reviewed and approved by Research Ethics Committee of the Evandro Chagas National Institute of Infectious Diseases. Written informed consent for participation was not required for this study in accordance with the national legislation and the institutional requirements.

## Author Contributions

AB-E and RZ-O participated in the study design. AB-E, GT, FA-S, and VR performed the experiments. AB-E, DF, MG-G, RA-P, and RZ-O designed the experiments, analysed and interpreted the data, and wrote the manuscript. All authors have contributed intellectually during the writing process, and have read and approved the final manuscript.

## Funding

This research was funded by Fundação de Amparo à Pesquisa do Estado do Rio de Janeiro (FAPERJ), grant number E-26/203.076/2016. RZ-O was supported in part by Conselho Nacional de Desenvolvimento Científico e Tecnológico [CNPq 302796/2017-7] and Fundação Carlos Chagas Filho de Amparo à Pesquisa do Estado do Rio de Janeiro [FAPERJ E-26/202.527/2019]. DF and RA-P received financial support from INI/Fiocruz (Programa Jovens Pesquisadores).

## Conflict of Interest

The authors declare that the research was conducted in the absence of any commercial or financial relationships that could be construed as a potential conflict of interest.

## Publisher’s Note

All claims expressed in this article are solely those of the authors and do not necessarily represent those of their affiliated organizations, or those of the publisher, the editors and the reviewers. Any product that may be evaluated in this article, or claim that may be made by its manufacturer, is not guaranteed or endorsed by the publisher.
